# Dreaming as mind wandering: evidence from functional neuroimaging and first-person content reports

**DOI:** 10.3389/fnhum.2013.00412

**Published:** 2013-07-30

**Authors:** Kieran C. R. Fox, Savannah Nijeboer, Elizaveta Solomonova, G. William Domhoff, Kalina Christoff

**Affiliations:** ^1^Department of Psychology, University of British ColumbiaVancouver, BC, Canada; ^2^Dream and Nightmare Laboratory, Center for Advanced Research in Sleep Medicine, Hôpital du Sacré-Coeur de MontréalMontréal, QC, Canada; ^3^Individual Studies, Université de MontréalMontreal, QC, Canada; ^4^Department of Psychology, University of California at Santa CruzSanta Cruz, CA, USA; ^5^Brain Research Centre, University of British ColumbiaVancouver, BC, Canada

**Keywords:** dreaming, mind wandering, default mode network, first-person report, spontaneous thought, neurophenomenology, memory consolidation, introspection

## Abstract

Isolated reports have long suggested a similarity in content and thought processes across mind wandering (MW) during waking, and dream mentation during sleep. This overlap has encouraged speculation that both “daydreaming” and dreaming may engage similar brain mechanisms. To explore this possibility, we systematically examined published first-person experiential reports of MW and dreaming and found many similarities: in both states, content is largely audiovisual and emotional, follows loose narratives tinged with fantasy, is strongly related to current concerns, draws on long-term memory, and simulates social interactions. Both states are also characterized by a relative lack of meta-awareness. To relate first-person reports to neural evidence, we compared meta-analytic data from numerous functional neuroimaging (PET, fMRI) studies of the default mode network (DMN, with high chances of MW) and rapid eye movement (REM) sleep (with high chances of dreaming). Our findings show large overlaps in activation patterns of cortical regions: similar to MW/DMN activity, dreaming and REM sleep activate regions implicated in self-referential thought and memory, including medial prefrontal cortex (PFC), medial temporal lobe structures, and posterior cingulate. Conversely, in REM sleep numerous PFC executive regions are deactivated, even beyond levels seen during waking MW. We argue that dreaming can be understood as an “intensified” version of waking MW: though the two share many similarities, dreams tend to be longer, more visual and immersive, and to more strongly recruit numerous key hubs of the DMN. Further, whereas MW recruits fewer PFC regions than goal-directed thought, dreaming appears to be characterized by an even deeper quiescence of PFC regions involved in cognitive control and metacognition, with a corresponding lack of insight and meta-awareness. We suggest, then, that dreaming amplifies the same features that distinguish MW from goal-directed waking thought.

“*The implication is that fantasy and dreams are part of a single continuing fantasy process which is subject to certain transformations imposed by physiological and stimulus events. It is unnecessary to sleep in order to generate dream-like ideation, and, apparently, it is unnecessary to be awake in order to produce relatively coherent, undream-like ideation*”

–Eric Klinger (1971, p. 57).

## Introduction

Dreaming and daydreaming (or “mind wandering”) seem to have had an enormous influence on human civilization through the ages: they are alleged to have inspired René Descartes's revolutionary view of the mathematical unity of nature (Baillet, 1691; Browne, 1977) and major scientific breakthroughs including discovery of the Benzene ring by Kekulé (Benfey, 1958), formulation of the periodic table by Mendeleev (Strathern, 2000), and Nobel prize-winning research on the chemical basis of neurotransmission by Loewi (1960)—to cite only a few examples. Psychological research into the subjective content of these states has revealed an intriguing, if less sensational, picture of dreaming and mind wandering (MW) as complex integrations of sensorimotor imagery, emotions, memories, and future planning, in which problem-solving can also occur (Domhoff, 2003). Yet these simultaneously mundane and exceptional mental states remain difficult to understand and study, in part because they are subjective and “spontaneous” in nature: undirected, unpredictable, and poorly characterized from both the personal and scientific perspectives.

Even after decades of scientific research, both behavioral and neurophysiological (reviewed in Hobson et al., 2000; Smallwood and Schooler, 2006; Klinger, 2008; Kussé et al., 2010; Schredl, 2010; Christoff et al., 2011; Gruberger et al., 2011; Zadra and Domhoff, 2011; Christoff, 2012), the sheer diversity of findings and perspectives on dreaming and MW can be overwhelming. MW has been characterized as an unwelcome detriment to professional (Smallwood et al., 2011) and educational (Smallwood et al., 2007) performance, as well as personal affect (Killingsworth and Gilbert, 2010), but has also been suggested to have an adaptive role in goal-directed planning (Christoff et al., 2009; Baird et al., 2011; Andrews-Hanna, 2012), deliberation on current concerns (Klinger, 1971, 2008), and creative insight (Baird et al., 2012). Views on dreaming similarly span a broad spectrum: dream mentation is considered by various researchers to be equivalent to brain delirium (Hobson, 1997) and schizophrenic psychosis (Solms and Turnbull, 2002), or to be entirely epiphenomenal (Flanagan, 1995). Others, however, have seen in dreaming a wellspring of individual growth and inspiration (Bulkeley, 2010), a source of creativity, insight and problem-solving (Schredl and Erlacher, 2007), an opportunity for emotional adaptation (Cartwright et al., 1998; Lara-Carrasco et al., 2009), and an expression and potential means of memory consolidation (Nielsen and Stenstrom, 2005; Wamsley and Stickgold, 2011).

Whereas specific beneficial (or conversely, disruptive) roles remain largely speculative, however, similarities between both the subjective and neurophysiological aspects of MW and dreaming have recently been explored in some detail (Pace-Schott, 2007, 2011; Christoff et al., 2011; Domhoff, 2011). In order to further address this question, we outline the general understanding of what dreaming and MW are, then discuss similarities in the subjective experience and neural basis of both states. Finally, we conduct and compare meta-analyses of positron emission tomography (PET) and functional magnetic resonance imaging (fMRI) studies of dreaming (rapid eye movement or “REM” sleep) and MW (DMN activity) in order to examine potentially overlapping neural substrates.

### What are dreaming and mind wandering?

“Dreaming” is usually understood as subjective mental experiences during sleep. Although most famously (and strongly) associated with REM sleep (Aserinsky and Kleitman, 1953; Dement and Kleitman, 1957), dream-like thought is also reported during other sleep stages (see Methods).

For several reasons, by “dreaming” we will generally be referring to subjective reports drawn from REM sleep: for one thing, the majority of “dream” reports have been elicited from REM sleep-stage laboratory awakenings; further, only REM sleep shows a particularly strong correlation with dream mentation (~80% of awakenings from REM sleep result in dream reports: Hobson et al., 2000). For the purposes of the present paper, then, “dreaming” refers to mentation reports from REM sleep.

“Undirected” thought is a similarly complex construct, and can be divided into several different categories (Christoff, 2012). “Mind wandering” (MW) and “stimulus-independent thought” (SIT), for instance, are typically defined as thinking that deviates from a particular task a subject is meant to be completing (McGuire et al., 1996; Mason et al., 2007; Christoff et al., 2009). “Spontaneous thought,” on the other hand, is characterized rather by its undirected, effortless nature—more akin to the everyday concept of “daydreaming” (Singer, 1966; Klinger, 1990; Christoff, 2012); no particular task, or deviation from it, is required. Subtle differences are apparent: MW, for example, might be initiated deliberately (as when a subject decides to “tune out” during a boring task) rather than being “spontaneous.” Nonetheless, these terms are often used interchangeably or with only minimal definition. Fluidity of terminology seems inevitable, however, in a relatively young field of inquiry (Christoff, 2012); moreover, the subjective content and neural basis of these states appear highly similar (compare, e.g., Singer and McCraven, 1961; Christoff et al., 2004, 2009; Stawarczyk et al., 2011). We therefore use these terms relatively interchangeably throughout this paper. MW, spontaneous thought, or daydreaming, then, all refer to subjective reports of undirected thoughts during wakefulness (whether deviating from, or in the complete absence of, a task).

### The default mode network (DMN) and REM sleep

Though specific neural correlates of both daydreaming and dreaming remain somewhat elusive, these mental states, and their associated subjective content, are strongly correlated with the “resting state” and REM sleep, respectively (Aserinsky and Kleitman, 1953; Dement and Kleitman, 1957; Maquet et al., 1996; Mason et al., 2007; Christoff et al., 2009; Andrews-Hanna et al., 2010; Vanhaudenhuyse et al., 2010; Christoff, 2012; Hasenkamp et al., 2012).

The default mode network (DMN) was discovered somewhat serendipitously as a pattern of brain *deactivations* associated with the difference between brain activity during a quiet, resting state (the typical baseline condition for early fMRI studies) and a goal-oriented, directed task (Raichle et al., 2001). Particular regions were consistently *more active* during “rest” than during goal-directed tasks of many kinds, suggesting a “default mode” network of regions active when a subject was “doing nothing” (Raichle et al., 2001; see Table 3 and Figure 2 for core regions of the DMN). It quickly became clear, however, that physical “rest” by no means implied mental inactivity. With no explicit task, subjects almost immediately engaged in spontaneous thought, including daydreaming, planning for the future, recalling memories, and so on (Gusnard et al., 2001). Subsequent research has tied the subjective experience of MW to core DMN regions (Christoff et al., 2004, 2009; Mason et al., 2007; Andrews-Hanna et al., 2010; Vanhaudenhuyse et al., 2010; Hasenkamp et al., 2012). Although regions beyond the DMN appear to also be recruited during MW (e.g., Christoff et al., 2009), the DMN still remains the most commonly used neural proxy for spontaneous thought (see also Methods).

**Figure 1:**
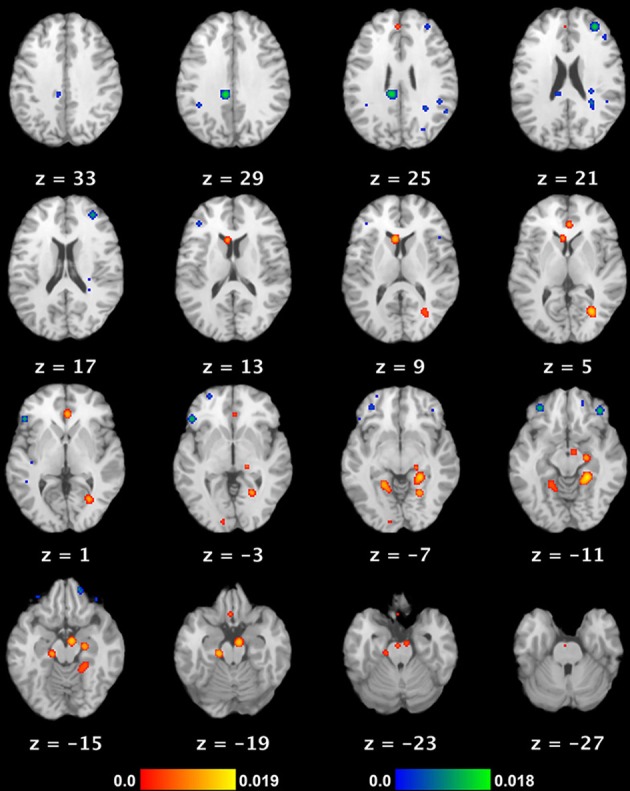
**Neural substrate of REM sleep vs. waking rest**. Significant meta-analytic clusters contributing to the neural substrate of REM sleep (as a proxy for dream mentation). Axial slices are displayed in Talairach space, with 3 mm skip. Color bars indicate likelihood that peaks represent actual peaks of difference at a given voxel. Activations (REM > waking rest) are in red-yellow, deactivations (REM < waking rest) in blue-green.

REM sleep is initiated by a network of cells in the pons and nearby portions of the midbrain (Siegel, 2011), but involves a widespread recruitment of higher cortical brain regions (see our meta-analytic results, below, for regions of this theoretical REM network: Table 2 and Figure 1). REM sleep recurs, in increasingly lengthy periods, approximately every 90 mins throughout the sleep cycle, overall constituting about 1.5–2 h of an average night of sleep. Whereas non-REM (NREM) sleep stages are generally characterized by deactivation of many regions as compared to wakefulness (e.g., Kaufmann et al., 2006), REM is unique in that many brain regions are clearly more active than during wakefulness (Table 2, Figure 1). REM also appears to be the most active state from the subjective point of view, with longer, more emotional, and more frequent dream mentation in REM than any other sleep stage (Hobson et al., 2000). REM therefore appears to be by far the best neural marker of dreaming, though it nonetheless remains problematic (see Methods).

### Subjective and neural similarities between dreaming and mind wandering

A number of similarities in the subjective experience of dreaming and MW have previously been noted (see Section First-person Reports of Content from Mind Wandering and Dreaming for a detailed overview). The possibility that the neural substrate of the DMN might be involved in, overlap with that of dreaming/REM sleep has also been raised (Fosse and Domhoff, 2007; Pace-Schott, 2007, 2011; Ioannides et al., 2009; Nir and Tononi, 2010), but these comparisons too have remained qualitative: a quantitative meta-analysis has yet to be applied to the question of the similarity in neural substrates between DMN/MW and REM sleep/dreaming. While major reviews and meta-analyses of the DMN have allowed for a tentative consensus regarding its neural basis (e.g., Buckner et al., 2008), a meta-analytic evaluation of brain activity during REM sleep has yet to be undertaken, making a direct comparison between brain activity in the two states difficult. The execution of such a meta-analysis of REM sleep was therefore a major goal of the present review.

### Aims of the present review and meta-analysis

Here we aim to build on prior qualitative comparisons of both the experiential and brain basis of dreaming and spontaneous thought with a more definitive, quantitative assessment of the similarity in brain activity. A strong reliance on first-person reports of subjective experience has guided much research on both MW and dreaming, and led to breakthroughs in the understanding of their respective neural correlates. Accordingly, we present a detailed discussion of first-person content reports from both states in Section First-person Reports of Content from Mind Wandering and Dreaming. We outline our methods of meta-analysis of functional neuroimaging data in Section Methods. In Section Neuroimaging of Mind Wandering and Dreaming: Meta-analytic Results, we meta-analyze results from functional neuroimaging (PET) studies of REM sleep (see Methods). We compare these results to an authoritative meta-analysis of DMN regions (Buckner et al., 2008) to determine to what extent the neural substrate of REM sleep overlaps with that of the DMN. Finally, we present a discussion of findings, limitations, and future directions, and propose a preliminary model of dreaming as “intensified” mind wandering.

## First-person reports of content from mind wandering and dreaming

Similarities in subjective content have been noted since the beginning of such research. For instance, the dreamlike nature of relaxed waking thought was documented in two early studies of what is now called MW, which were carried out in a sleep laboratory using EEG to monitor wakefulness. In both studies, participants were randomly asked to report anything that was going through their minds at the time of the probe. In the first study, Foulkes and Scott (1973) found that 24% of thoughts could be categorized as visual, dramatic, and dreamlike. In a replication study, Foulkes and Fleisher (1975) discovered that 19% of reports were dreamlike.

The qualitative characteristics of dreaming have been intensively studied over the past century, yielding a considerable body of research from which some firm conclusions can be drawn regarding subjective content. Though qualitative data on the content of MW is not nearly as comprehensive, a tentative overview is nonetheless possible. Although a comprehensive review of the literature is beyond the scope of this article, we highlight consistent findings regarding the subjective content of dreaming and MW. We focus on similarities in subject matter across several key areas, including sensory, emotional, fanciful, mnemonic, motivational, and social aspects, as well as addressing the presence or absence of cognitive control and metacognition. Various disparities and inconsistencies are addressed here, as well as in the Discussion.

### Sensory aspects

The broadest similarity between dreaming and MW is perhaps also the most basic: the sensory building blocks of spontaneous thought in both waking and dreaming are overwhelmingly visual and auditory (though experiences in other sensory modalities are by no means precluded).

#### Dreaming

The largely audiovisual nature of dreaming was noted over two millennia ago by Artemidorus in his *Oneirocritica* (Harris-McCoy, 2012) and has been often replicated in contemporary research. For instance, a recent review of dream content (Schredl, 2010), based on more than 4000 dream reports from both laboratory awakenings and home dream diaries, found that visual content was present in 100%, and auditory content in ~57%, of all reports (Table 1). Other sense modalities (tactile, olfactory, gustatory, and nociceptive experiences), by contrast, were present in ~1% or less of all reports. Indeed, the next most prominent modality after vision and audition was the vestibular sense: ~8% of reports contained experiences of flying, floating, acceleration, etc. (Schredl, 2010). Intriguingly, a comparison with studies of dream reports from more than a century ago shows a very similar trend: in the late nineteenth century, dream reports also almost always featured visual elements, followed by auditory imagery as the next most dominant aspect, and with the remaining senses accounting for very small percentages (~1–7%) (Schwartz, 2000). This suggests that the sensory aspects of dreaming may be consistent cross-culturally (or at least, cross-temporally).

**Table 1:** **Sensory perception in dreaming**.

**Modality**	**Frequency (% of reports)**
Visual	100
Auditory	57
Vestibular	~8
Tactile	~1
Gustatory	~1
Olfactory	~1
Pain	~1

Based on Schredl (2010).

The apparent predominance of audio-visual content in dreams may underestimate other sensory modalities, however. A number of studies sampling other sensory data revealed that, when prompted specifically for sensations such as pain (Nielsen et al., 1993; Raymond et al., 2002; Solomonova et al., 2008) or bodily orienting movements (Solomonova et al., 2008), participants often reported more information. To our knowledge, similar targeted sensory-content probes have not yet been undertaken during MW, precluding a more detailed comparison.

#### Mind wandering

Content findings from mind wandering are not usually directly comparable, since MW researchers have tended to focus on the intensity (rather than the prevalence) of audiovisual imagery, but available evidence suggests similar trends. For example, factor analysis of nearly 1500 experience reports found that visual and auditory intensity are two of eight dimensions significantly characterizing spontaneous thoughts (Klinger and Cox, 1987). A more recent study similarly found a very high prevalence of self-reported visual and auditory imagery during spontaneous thoughts (mean ratings of 4.22 and 4.02, respectively, on a 7-point Likert scale) (Stawarczyk et al., 2011). Along these lines, a recent review concluded that the average spontaneous thought is moderately visual, contains at least some sound, and is very likely (74% of reports) to contain some form of interior monolog or “self-talk” (Klinger, 2008).

### Positive and negative emotionality

#### Dreaming

It appears that most dreams (~70–75% or more in adults) contain some emotion, though affect in dreams may not always be particularly strong, or appropriate to the context (see Domhoff, 2011, for a discussion). A number of studies have found a relative predominance of negative emotions in dreams, particularly when dreams are scored by judges rather than by dreamers (see Schredl, 2010, for a review). Other studies, however, have found a balance of emotions in REM sleep dream reports, and one study (Fosse et al., 2001) found that joy/elation was in fact the most frequently reported emotion. An interesting study directly compared self-reports of dreaming vs. waking events, finding that negative emotion (particularly fear) was more prevalent during dreaming, and positive emotions more common in waking (Nielsen et al., 1991).

It may be, however, that more intense and negatively toned dreams are better remembered, and thus over-reported. Additionally, sampling techniques (e.g., laboratory awakenings vs. home dream journals) may contribute to differences in findings. Irrespective of these differences and methodological limitations, however, it is evident that both positive and negative emotions are ubiquitous during dreaming.

#### Mind-wandering

Though not yet extensively studied, emotion appears to be similarly ubiquitous during MW. One recent study, for instance, involving thousands of reports, found that the majority (69%) of spontaneous thought reports involved emotion (positive emotion in 42.5% of reports, negative emotion in 26.5%), whereas only 31% of reports were reported to be emotionally neutral (Killingsworth and Gilbert, 2010). Though data are generally lacking, it is interesting to note that, in contrast to dreaming, positive emotion appears to predominate during waking MW, and that many more waking spontaneous thoughts appear to be characterized by relatively flat (neutral) affect. Also of interest is that the temporal focus of MW content appears to be more directed toward the past when negative mood has been experimentally induced (Smallwood and O'Connor, 2011).

### Implausibility and bizarreness

Though the typical spontaneous thought or dream is a relatively plausible simulation or elucidation of past memories, current events, or future plans, generally in line with the current concerns of the subject (see “Motivational Aspects,” below), nonetheless implausible and bizarre elements are common to both states—though their precise frequency remains disputed (Snyder, 1970; Dorus et al., 1971; Zadra and Domhoff, 2011). Examples are physically impossible or socially unlikely situations, fanciful locales and characters, large discontinuities of time and/or space, and so on.

#### Dreaming

Depending on scoring criteria, it has been estimated that between 32% (Schredl, 2010) and 71% (Stenstrom, 2006) of dream reports feature bizarre or impossible elements. Despite widely varying estimates, however, there is general agreement that bizarre, incongruous or impossible elements are features of at least a substantial proportion of dreams. Differences in precise estimates are likely due to differing scoring procedures, as well as differences between dreamer- or judge-rated scores.

#### Mind-wandering

Though many MW episodes contain relatively realistic simulations of plausible events in the external world, nonetheless a substantial number (~20% of reports) contain elements that are bizarre, implausible, or fanciful (defined as “departing substantially from physical or social reality”) (Klinger and Cox, 1987; Kroll-Mensing, 1992; Klinger, 2008). A more recent study has provided a general replication of earlier results: analyzing thousands of thoughts reported by 124 subjects, Kane et al. (2007) found that the average thought during MW contained a moderate level of fantasy (a mean of 3.77 on a 7-point scale).

In a rare study examining both waking fantasy and dream reports in the *same* 12 subjects, Williams et al. (1992) found that bizarre elements were about twice as prevalent in dreams vs. waking spontaneous thought. In a similar vein, dream and daydream bizarreness have been studied in relation to “thick” vs. “thin” boundaries (Kunzendorf et al., 1997): though thin boundary personality was associated with more bizarre dreams and daydreams than thick boundary, dreams were scored more bizarre than daydreams across both personality types.

### Mnemonic features: contributions of episodic and semantic memory

Both dreaming and MW draw on episodic and semantic memory sources as building blocks for novel subjective experiences. In this section we discuss the prevalence of past-oriented thoughts during both wakefulness and dreaming, and the potential contributions of both episodic and semantic memory to these states.

#### Dreaming

There is an intriguing literature suggesting that sleep, especially NREM sleep, may have a role in memory consolidation (Walker and Stickgold, 2006; Born and Wilhelm, 2012), including specific roles for REM sleep in consolidation of procedural (Smith et al., 2004) and emotional episodic (Nishida et al., 2009; Groch et al., 2013) memories. A dynamic model of sleep-dependent memory consolidation and reconsolidation has recently been proposed, suggesting a complex relationship between sleep stages, memory types and their contribution to cognitive stability, flexibility and brain plasticity (Walker and Stickgold, 2006, 2010).

It is now well documented that dream content borrows from both temporally proximal and distal memories (Nielsen and Stenstrom, 2005). The most proximal memories (those from the previous day) are generally known as “day residue” (Freud, 1908), whereas the recurrence of elements 5–7 days following an experience is referred to as the “dream-lag” effect (Nielsen and Powell, 1989). Personally relevant and emotionally salient events appear to manifest themselves in dream content as day residue and dream lag effects, but can also surface many years after initial encoding (Grenier et al., 2005). The presence of emotional and personally relevant content in dreams may be related to the fact that emotional and impactful events are preferentially consolidated in memory (McGaugh et al., 2002; Nishida et al., 2009). While dreaming contains clear episodic autobiographical elements, memories only rarely get “replayed” in dream content (~1–2% of reports: Fosse et al., 2003).

#### Mind-wandering

MW appears to involve roughly equal percentages of thoughts about the past and future (Fransson, 2006), though some studies suggest a “prospective bias” toward future-oriented thoughts (Smallwood et al., 2009; Andrews-Hanna et al., 2010; Stawarczyk et al., 2011), and also a past-bias inducible by negative mood (Smallwood and O'Connor, 2011). Overall, however, it is clear that memories, particularly episodic ones, play a large role in spontaneous thought. Many studies have reported a high prevalence (~20% or more of reports) of past-focused MW (Fransson, 2006; Smallwood et al., 2009; Andrews-Hanna et al., 2010; Smallwood et al., 2011). Indeed, one of the first studies to explore “resting state” activity using PET noted the similarities between such activity and episodic memory recall, as well as the fact that subjective reports of “rest” actually involved a large amount of past recollection and future planning (Andreasen et al., 1995). Similar to dreaming, memories incorporated in waking MW tend to be of emotional and personally relevant material, and are often related to people's current concerns (see section below on “Motivational Aspects”).

In summary, dreaming and MW both contain specific traceable episodic and semantic memory sources, but very rarely reproduce memories in their entirety. Rather, memories tend to reappear in novel, re-contextualized thoughts and scenarios (Nielsen and Stenstrom, 2005).

### Motivational aspects: current concerns

Reports from both dreaming and MW show a strong proclivity to reflect the ongoing concerns of subjects, as well as elements of anticipating and planning for the future.

#### Dreaming

A wealth of data supports the notion that dreaming reflects ongoing waking concerns, desires, and experiences, in line with the “continuity hypothesis” of dreaming and waking mental activity (see, e.g., Domhoff, 1996, Ch. 8). For example, transient stressful situations, such as divorce (Cartwright et al., 1984) and grief (Kuiken et al., 2008) are also often present in dream reports in a general form.

Although dream content is often found to be thematically and emotionally consistent with the waking state of the dreamer, certain activities prevalent in waking are only rarely found in dreams. These include cognitive activities such as reading, writing, and using a phone or a computer (Schredl, 2000).

#### Mind-wandering

Similar to dreaming, the content of waking MW also centers heavily on subjects' current concerns (Klinger and Cox, 1987; Klinger, 2008; Andrews-Hanna, 2012).

Further, when the temporal focus of MW is examined, a large percentage (~40% in one recent study: Andrews-Hanna et al., 2010) of spontaneous thoughts center around the present time ±1 day, supporting the notion that MW strongly involves current concerns and experiences. Future-oriented thought is also incredibly common during MW (Smallwood et al., 2009; Andrews-Hanna et al., 2010; Stawarczyk et al., 2011), further supporting a role for MW in future-planning and potentially problem-solving. Intriguingly, in one of the few neuroimaging studies to directly examine periods of MW, MW was associated with activations not only in the DMN but also in key executive prefrontal areas, including the dorsal anterior cingulate cortex and dorsolateral prefrontal cortex (Christoff et al., 2009). Such results are consistent with the prevalence of current concerns and unresolved issues in first-person content reports, and may reflect an ongoing (if unconscious) effort to address them (Christoff et al., 2009; see also Discussion).

### Imagined social interaction

#### Dreaming

Similar to waking life, dreaming is nearly always organized around interactions with others. Most dreams include other characters in some kind of relationship with the dreamer, or a generalized social situation (Hall and Van de Castle, 1966; Nielsen et al., 2003; Schredl et al., 2004; Zadra and Domhoff, 2011). Social interactions in dreams follow a multitude of patterns, including threatening (Valli et al., 2005) and otherwise emotionally-charged situations (Cartwright et al., 1984). Occasionally, recognizable dream characters may change appearance or appear as a generalized entity, fused with features of other individuals. Also of interest is the prevalence of “mentalizing” or use of “theory of mind” in dreaming—i.e., thinking about others' thoughts, emotions and motivations (even though the “others” are of course merely imagined) (McNamara et al., 2007). In general, meaningful interactions with others may be one of the key factors guiding the progression of the dream narrative.

#### Mind wandering

First-person reports of MW often involve imagined social interactions with others, as well as thoughts about the intentions and beliefs of other people (Klinger, 2008). This has led to the general notion that “mentalizing” (i.e., thinking about the thoughts and minds of others) and the consideration of hypothetical social situations may be key components of spontaneous thought (Buckner et al., 2008; Andrews-Hanna, 2012). Supporting this idea, numerous studies have found that brain activity underlying “theory of mind” and mentalizing overlaps significantly with DMN regions (see Buckner et al., 2008, for a review).

### Cognitive control and metacognition

#### Dreaming

A singular aspect of dreams is the seemingly total lack of metacognitive awareness in the dream state. One experiences a complex simulation of oft-bizarre experiences, but without the overt capacity to reflect on the bizarre state of affairs the mind and body are actually in see, e.g., Rechtschaffen (1978). Intriguingly, it appears that well-trained, or talented, individuals can develop metacognitive awareness of the dream state, becoming “lucid” in the dream and sometimes even directing its course and content (Dresler et al., 2012). The exceptional nature of “lucid” dreaming, however, serves to prove the rule of the general lack of control and metacognitive awareness in ordinary dreaming, a characteristic likely attributable to the deactivation of numerous prefrontal cortical regions during REM sleep (see our results in Table 2 and Figure 1; also Hobson et al., 2000; Muzur et al., 2002).

**Table 2:** **Core cortical components of the neural network underlying REM sleep**.

**Region**	**Cluster size (mm^3^)**	**Talairach coordinates (*x*, *y*, *z*) [BA]**
**ACTIVATIONS (REM > WAKING REST)**		
**Cortical regions**		
Medial prefrontal cortex	368	2, 32, 2 [Area **24**]
Posterior cingulate cortex/lingual gyrus	656	28, −66, 4 [Areas 19, **30**]
Parahippocampal cortex	1088	24, −40, −10 [Areas **36**, 37]
	416	−16, −26, −18 [Area 35]
Parahippocampal/entorhinal cortex	104	18, −30, −6 [Areas **28**, **35**]
Posterior parahippocampus/lingual gyrus	496	−18, −50, −8 [Area 19]
	352	22, −58, −6 [Areas 19, **36**]
Entorhinal cortex/hippocampus	360	22, −18, −14 [Areas **28, 35**]
**Subcortical regions**		
Pons/midbrain	688	8, −14, −18
Caudate nucleus	472	−6, 16, 10
**DEACTIVATIONS (REM < WAKING REST)**		
**Cortical regions**		
Mid/posterior cingulate	752	−8, −34, 28 [Area **23**]
Rostrolateral prefrontal cortex	456	32, 44, 20 [Area 10]
Inferior frontal gyrus	296	−46, 26, −2 [Areas 47, 45]
Orbitofrontal cortex	256	−32, 38, −10 [Area 11]
	224	38, 36, −12 [Area 11]
	120	18, 46, −14 [Area 11]
Superior longitudinal fasciculus	176	28, −42, 20

**Figure 2:**
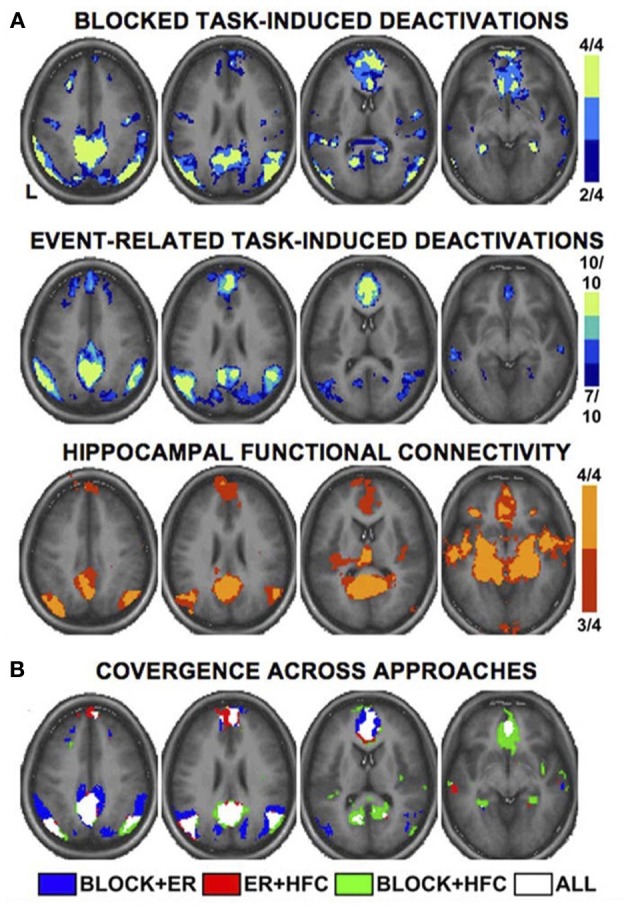
**Multiple fMRI methods defining the default mode network**. Key cortical areas contributing to the default mode network, as revealed by distinct fMRI methods and study designs. Data are based on a meta-analysis by Buckner et al. (2008) that included 4 blocked design fMRI studies, 10 event-related fMRI studies, and 4 studies of functional connectivity. Colors for each panel of images denote the number of studies finding significant effects at a given voxel (see color bars and numeric labels at right). Note the similarity in the pattern of regions recruited, regardless of method **(A)**, and the strong convergence across all methods **(B)**. Reproduced with permission from Buckner et al. (2008); originally adapted from Shannon (2006). ER, event-related; HFC, hippocampal functional connectivity.

Peak cortical foci of likely activation and deactivation from a meta-analysis of all functional neuroimaging (PET) studies of REM sleep compared to a baseline of waking rest. Notably, every cortical cluster of activation overlaps (convergences in *bold* font) with a core component of the DMN, except for one cluster in left lingual gyrus [Area 19] (compare with Table 3 and Figure 2). Conversely, significant clusters of deactivation overlap with DMN regions in only one case out of seven. The cluster labeled as in superior longitudinal fasciculus is approximate only. BA, Brodmann area; DMN, default mode network; PET, positron emission tomography; REM, rapid eye movement.

#### Mind wandering

A lack of *explicit* goals, and an unawareness that one is even daydreaming or has deviated from the task at hand, are typical of MW (Schooler et al., 2011). But although MW tends to be less characterized by intentional thought and self-reflective awareness, this is not always the case. A recent study from our group, for instance, found that subjects who were probed at random intervals reported being unaware that they had been mind wandering about half (45%) of the time. One's impression of the “controllability” of a segment of MW also varies widely, from a sense of being able to end it at any time, to being completely absorbed in and swept along by a daydream (Klinger, 1978, 2008; Klinger and Cox, 1987; Kroll-Mensing, 1992; Klinger and Kroll-Mensing, 1995). Collectively, these results suggest that cognitive control and metacognitive awareness in MW lie somewhere between the relative lucidity and self-reflectiveness of normal waking thought and behavior, and the near-total lack of control and metacognitive nescience characteristic of regular (i.e., non-lucid) dreams. See the Discussion for an elaboration of this theme.

## Methods

### Study selection for neuroimaging meta-analysis

#### Dreaming

Though the two phenomena have often been seen as synonymous since Aserinsky and Kleitman's discovery of the association between the REM sleep and dreaming (Aserinsky and Kleitman, 1953), dream-like mental activity occurs in all sleep stages (Nielsen, 2000), including briefly at sleep onset (NREM1) (Mavromatis, 1987; Hori et al., 1994; Nielsen et al., 2005) as well as in NREM2 (Antrobus et al., 1995; Fosse et al., 2004), particularly later in the night (Cavallero et al., 1992). Mentation from NREM3/4 sleep, also known as Slow Wave Sleep (SWS), has also been reported, albeit more rarely (Cavallero et al., 1992). In line with the markedly different patterns of brain activity throughout the sleep cycle (Kaufmann et al., 2006), the length, bizarreness, and emotionality of dream reports from various sleep stages appear to differ significantly, though these disparities remain controversial, with some researchers arguing that the important issue is level of cortical activation, not sleep stage (Antrobus et al., 1995; Cicogna et al., 1998; Foulkes, 1999).

There is also strong evidence from neuropsychological lesion work that the neural mechanisms underlying REM sleep and dreaming are doubly dissociable (Solms, 1997, 2000, 2011; Oudiette et al., 2012). Nonetheless, we use neuroimaging studies of REM sleep *only* as a neural proxy for the brain basis of dreaming in the present study, for several reasons: (1) NREM1, far from being a uniform state, can be subdivided into at least 8 sub-stages (Hori et al., 1994). Hallucinatory, dream-like mentation is only strongly associated with particular sub-stages, especially those with strong EEG theta rhythms (Hori et al., 1994). To our knowledge, however, no neuroimaging study has yet examined these brief epochs in isolation. Data collapsed across all phases of NREM1, then, is an unsuitable neural marker for dream-like mentation. (2) Of the few functional neuroimaging studies of sleep, NREM2 sleep is rarely explicitly divided into early and late stages based on the ultradian changes in its EEG microarchitecture (Roth and Roehrs, 2000). Since only late NREM2 is even moderately correlated (~0.40) with dream mentation (Nielsen, 2000), data collapsed across all phases of NREM2 (which is all that is currently available) is likewise unsuitable. (3) Despite the apparent dissociability of REM sleep and dreaming, the two remain extremely highly correlated, with roughly 70–90% of awakenings from REM sleep yielding dream reports (~83% on average: Nielsen, 2000). So while other sleep stages clearly give rise to dream-like mentation, we contend that REM is “the best and most frequent trigger” for dreaming (Domhoff, 2005; p. 5) and is therefore the best objective neural indicator of strong dream mentation at the present time.

We therefore reviewed all functional neuroimaging (PET or fMRI) studies of REM sleep to date (14 studies; Table A1). In order to minimize the confounding effects of various tasks and baseline conditions, only studies employing a baseline of resting wakefulness (either pre- or post-sleep) were included. A total of 6 studies were included, and 8 excluded, from the meta-analysis (detailed in Table A1). Other reasons for exclusion included the addition of extraneous factors (e.g., auditory stimulation during REM sleep), inclusion of clinical populations, failure to provide information for peak foci of activation, or lack of an appropriate baseline (e.g., studies comparing REM sleep during phasic rapid-eye-movement events with regular tonic REM sleep).

#### Mind wandering

Very few papers to date directly examine periods of mind wandering vs. non-mind wandering (Christoff et al., 2009; Vanhaudenhuyse et al., 2010; Hasenkamp et al., 2012). Though numerous other studies have addressed mind wandering indirectly, they tend to assume an *a priori* link between DMN activity and MW (e.g., Mason et al., 2007; Andrews-Hanna et al., 2010). It appears, however, that this assumption is at least somewhat warranted: Christoff et al. (2009) and Hasenkamp et al. (2012) indeed found stronger activity in major hubs of the DMN (as well as in other regions beyond the DMN) during MW, and Vanhaudenhuyse et al. (2010) similarly found that self-reported intensity of internally-directed thinking correlated with stronger activity in pre-defined DMN regions of interest (ROIs) (e.g., anterior and posterior cingulate cortices, as well as parahippocampal cortices). Direct meta-analysis of regions active in MW was not executed, however, because only Christoff et al. (2009) have used normal subjects, whole-brain (WB) analyses, and direct, online measures of MW. Though Hasenkamp et al. (2012) used direct, online MW measures and WB (vs. ROI) analyses, they exclusively employed a specialist population (long-term meditation practitioners); Vanhaudenhuyse et al. (2010) also used online MW measures and normal subjects, but employed *a priori* ROIs in their analyses. These considerations render the comparability of the three studies questionable, and a meta-analysis of MW-related brain activations premature. As with REM sleep and dreaming, then, we utilize DMN activity as an imperfect neural marker for spontaneous thought/MW/daydreaming.

#### Identifying core regions of the default mode network

As a meta-analysis of MW brain activations was precluded by the above considerations, we consulted a recent comprehensive review of DMN functional neuroanatomy (Buckner et al., 2008) in order to highlight cortical regions thought to be key hubs of the DMN (and by extension, spontaneous thought). There are numerous ways of determining DMN activity: early studies used blocked time periods of task vs. rest, whereas more recent studies have generally used event-related study designs or functional connectivity analysis. Notably, there is high convergence across these several techniques (see Figure 2). The final summary of key DMN regions that we employ here (Table 3) involves the combination and convergence of data from all three methods, and was gleaned by Buckner et al. (2008) from the review of 18 data sets employing hundreds of subjects.

**Table 3:** **Core cortical components active in the default mode network**.

**Region**	**Approximate brain areas (BA)**
Ventromedial prefrontal cortex	24, 10 m/10 r/10 p, 32 ac
Dorsal medial prefrontal cortex	24, 32ac, 10p, 9
Posterior cingulate/retrosplenial cortex	29/30, 23/31
Inferior parietal lobule	39, 40
Lateral temporal cortex	21
Hippocampus	–
Parahippocampus	35, 36
Entorhinal cortex	28, 34

Key cortical brain structures contributing to human default mode network activity. Note that most (6 of 8) components of the DMN overlap with regions that are activated during REM sleep (Table 2), with the exceptions of the inferior parietal lobule and lateral temporal cortex. Adapted from Buckner et al. (2008). BA, Brodmann area.

#### Activation likelihood estimation (ALE) meta-analysis

We used a quantitative, random-effects meta-analytic method known as activation likelihood estimation (ALE) (Turkeltaub et al., 2002; Laird et al., 2005; Eickhoff et al., 2009, 2012) implemented in the software program GingerALE 2.1 (San Antonio, TX: UT Health Science Center Research Imaging Institute). The most recent ALE algorithm tests for above-chance clustering of peak foci from different experiments included in the meta-analysis (Eickhoff et al., 2009, 2012) by comparing actual activation foci locations/clustering with a null distribution created by distributing the same number of foci randomly throughout the brain, through several thousand iterations. Included activation foci were smoothed using a full-width half maximum (FWHM) Gaussian kernel dependent on the sample size (subjects) of the experiment from which foci were drawn [larger sample -> smaller smoothing kernel—empirically determined by (Eickhoff et al., 2009, 2012)]. Resulting statistical maps show clusters where convergence between activation foci is greater than would be expected by chance (i.e., if foci from each experiment were distributed independently).

For REM sleep, we meta-analyzed a total of 67 foci drawn from 6 studies (Table A1), which yielded 17 meta-analytic results (Table 2; 10 activations, 7 deactivations). Statistical maps were thresholded using a false discovery rate (FDR—Genovese et al., 2002) of *q* = 0.05 and a cluster threshold of *k* = 100 mm^3^. To display results, we used template brain images from GingerALE 2.1 displayed in the “Mango” software package (San Antonio, TX: UT Health Science Center Research Imaging Institute). No ALE meta-analysis of MW was undertaken (for reasons given in “Study Selection,” above).

## Neuroimaging of mind wandering and dreaming: meta-analytic results

### ALE meta-analysis of REM sleep

We observed 8 significant cortical clusters of activation (REM sleep > waking rest) associated with REM sleep, as well as 2 subcortical clusters in the brainstem (pons) and caudate nucleus (Table 2 and Figure 1). Of the 8 cortical clusters, 7 overlapped with key regions of the DMN (Table 2: convergences in bold font; compare with Table 3). We also observed 7 significant cortical clusters of deactivation (REM sleep < waking rest). Except for one area of overlap with the DMN in the mid/posterior cingulate cortex, almost all deactivations were in prefrontal areas.

### Core cortical components of the default mode network

We identified 8 core cortical regions of the default mode network (Table 3 and Figure 2) based on a recent authoritative review (Buckner et al., 2008; see also Methods).

## Discussion: the stream of (spontaneous) thought and its functions

“Imagery [i.e., spontaneous thoughts and fantasies] thus needs to be seen within this context–it is not simply produced under conditions of demand by tasks of learning or recall, but it almost continuously emerges into consciousness, probably as a feature of the very nature of the brain's function and of man as a plan-making organism”—Singer and Antrobus (1972, p. 176–177).

The appellation “daydreaming,” often used interchangeably with “mind wandering,” highlights the folk psychological similarity between MW and dreaming evident even in our language. Here we have provided evidence that both quantitative meta-analysis and qualitative comparisons support this ostensibly facile analogy. Our results suggest significant similarities in both the subjective content and neurophysiological signatures of these two apparently distinct states, amplifying observations and theoretical accounts of our own (Christoff et al., 2011; Domhoff, 2011) and others (Pace-Schott, 2007, 2011).

The idea that dreaming and MW may lie on a single continuum has a number of precedents. Freud (1908), for instance, saw dreams, daydreams and creative endeavors as reflections of the same underlying processes. More recently, we have explored the idea that dreaming may share the same associative mechanisms and recruit the same neural networks (particularly the DMN) as daydreaming (Christoff et al., 2011; Domhoff, 2011). Others have also proposed an uninterrupted mental continuum between very focused waking thought, waking MW, and fully immersive dreaming (Hartmann, 1996; see also Windt, 2010). Below, we expand on this idea of a continuum in our discussion of our qualitative and meta-analytic results. We also address limitations of the present meta-analysis, potential functions of spontaneous thought in both waking and dreaming, and future directions.

### Meta-analysis of cortical activity during REM sleep

To our knowledge, the present paper is the first to conduct a quantitative meta-analysis of functional neuroimaging studies of REM sleep. Based on data from six studies of essentially “pure” REM sleep (no extraneous stimuli or tasks, healthy non-clinical populations, comparison to waking baseline), we found 10 meta-analytic clusters of significant activation (REM > waking rest). As noted by the authors of the original studies, activated regions are highly consistent with the subjective aspects of dreaming. Clusters were observed in numerous high-level visual areas, such as the parahippocampal place area, fusiform gyrus, and lingual gyrus, consistent with the ubiquitous, immersive visual imagery characteristic of dreams. Regions implicated in long-term and episodic memory, as well as in imagining future scenes and situations (Schacter et al., 2007), are also active, including parahippocampal cortex, hippocampus, and entorhinal cortex. Finally, multiple clusters were observed in mPFC regions, which, most relevant to the present results, have been strongly implicated in self-referential thought and affective decisions (Raichle et al., 2001; Buckner et al., 2008; Andrews-Hanna et al., 2010). We also found several (7) clusters of deactivation, mostly in the frontal lobe—consistent with prior accounts (e.g., Muzur et al., 2002).

### Overlapping and non-overlapping patterns of brain activity in the DMN and REM sleep

When we compared our meta-analytic results for REM sleep to core regions of the DMN, we found substantial overlap. Specifically, of the 8 significant cortical clusters of activation identified in our ALE meta-analysis of REM sleep, all but one overlapped to at least some extent with core regions of the DMN. The most complete overlap is apparent in regions of mPFC and medial temporal lobe (MTL) structures, including parahippocampal, hippocampal, and entorhinal cortices (Table 2 and Figure 1). Importantly, other sleep stages show mostly deactivations compared to waking baselines, and generally in regions outside the DMN (e.g., Kaufmann et al., 2006). This suggests that the observed overlap with the DMN is not common to all sleep stages, but specific to REM sleep—the only sleep stage truly reliably associated with dream mentation.

The overlap is of course far from perfect (compare Table 2 and Figure 1 with Table 3 and Figure 2). Several regions *beyond* the DMN are evident in our results (fusiform gyrus, parahippocampal place area [PPA], and lingual gyrus). Conversely, several regions of the DMN are represented poorly (posterior cingulate cortex [PCC]) or not at all (inferior parietal lobule [IPL], lateral temporal cortex [LTC]) in our tentative REM sleep map. The most easily explained discrepancy is that numerous REM clusters extend beyond DMN regions to include cortical regions well known to be involved in high-level visual processing, such as the fusiform gyrus, PPA, and lingual gyrus. Such results are consistent with the highly visual nature of dreaming, and with our hypothesis (see below) that dreaming can be considered an intensified version of spontaneous waking thoughts (which are only moderately visual in nature—see Section First-person Reports of Content from Mind Wandering and Dreaming). Another discrepancy is in PCC. REM sleep meta-analysis revealed a large (656 mm^3^) cluster of activation in the area of the right PCC, but this cluster extended largely into the lingual gyrus (BA 19), and was more lateral than typical activations in DMN (e.g., Buckner et al., 2008) and during MW (e.g., Christoff et al., 2009). We also found a large (752 mm^3^) cluster of *de*activation in the area of the mid/posterior cingulate cortex. Further, the IPL was not observed at all in our meta-analytic REM sleep results.

Due to their rich reciprocal anatomical connections and strong functional connectivity with MTL structures, the PCC and IPL have been hypothesized to be involved in accessing episodic/autobiographical memories during spontaneous thought (Andrews-Hanna et al., 2010). These parietal regions may direct attention to such memories and make them available to higher cortical (e.g., prefrontal) regions, whereby they reach conscious awareness. If tenable, this putative role for PCC and IPL in spontaneous thought may in part explain the observed discrepancies in brain activation between REM sleep and the DMN. Though dreaming clearly draws on both long-term and recent memories (Section First-person Reports of Content from Mind Wandering and Dreaming), dream mentation almost never involves replay of particular episodic memories (Fosse et al., 2003). Moreover, the general lack of self-knowledge in dreams and the frequent failure to note abnormalities that an intact memory might easily notice (such as the appearance of deceased relatives), are well known phenomena. Finally, dreams are notoriously difficult to recall, even with regular practice, and especially after any significant delay. All the above considerations are consistent with a general disconnect during dreaming between highly active memory centers in the MTL and relatively quiescent hubs in the PCC and IPL of the parietal lobe.

Another possibility is that DMN regions we failed to detect in our meta-analysis (in particular, IPL and LTC) *are* indeed active during REM sleep, but are simply *no more* active than during waking rest—the baseline condition with which REM sleep was compared. Because studies of REM sleep have relied on simple contrasts (REM > waking rest), these regions could be just as active during REM sleep as during waking rest (and therefore, presumably, spontaneous thought). The lack of *significantly greater* activity, however, would prevent their detection either in the original REM sleep studies or in our meta-analysis (though this would not explain the cluster of deactivation we observed in mid-PCC). At present, available data cannot address this possibility, but one option for future research would be to carefully examine functional connectivity between regions active in REM sleep, to determine whether other areas (possibly IPL and LTC) are implicated. This strategy has been used to further explore regions involved in the DMN, and has led, e.g., to the conviction that, despite earlier ambiguity, medial temporal lobe structures are indeed a critical component (Buckner et al., 2008).

Aside from comparing overlap between regions key to both REM sleep and DMN activity, also of interest is the converse comparison: examining brain regions unrelated to dreaming and their potential overlap (or lack thereof) with DMN areas. In an exhaustive study relating brain lesion locus to dreaming in 332 neuropsychological patients, Solms (1997) found that 200 patients reported no changes in dream mentation. In support of our central hypothesis, lesions among these patients were primarily in sensorimotor cortices and dorsolateral PFC (Solms, 1997), none of which appear to be key to REM sleep (Table 2) or DMN functioning (Table 3).

**Figure 3:**
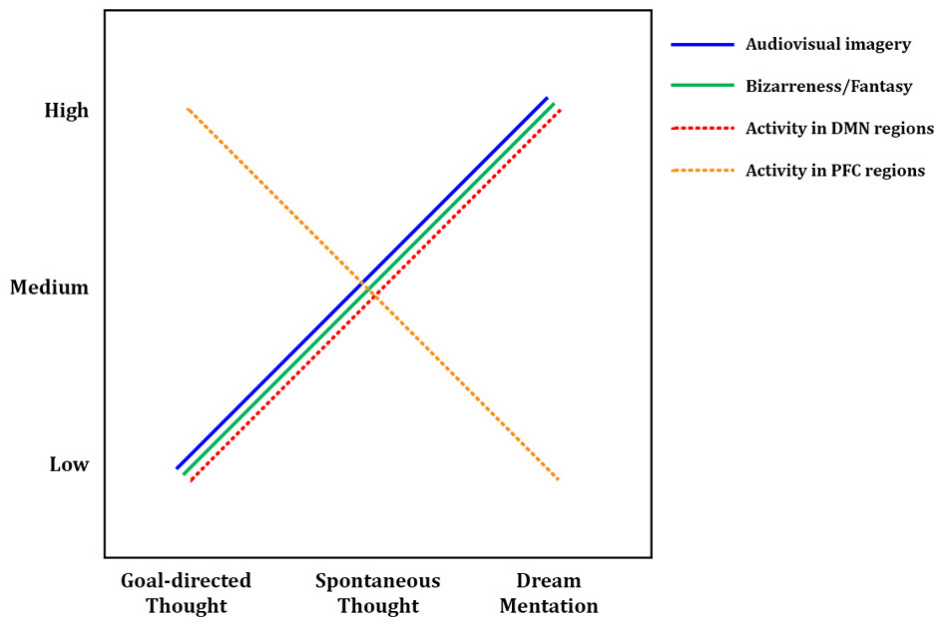
**Tentative model of dreaming as intensified mind wandering**. A preliminary model of dreaming as an intensified version of waking mind wandering. Intensity of audiovisual imagery, number of bizarre or implausible elements, and activity in DMN regions all appear to increase from waking, goal-directed thought, through waking spontaneous thoughts, to dream mentation. The opposite trend may hold for activity levels in prefrontal executive regions such as anterior cingulate cortex and dorsolateral prefrontal cortex, which are highly active in goal-directed waking thoughts and tasks, only somewhat active during mind wandering (Christoff et al., 2009), and mostly quiescent during dreaming/REM sleep (Table 2). Solid lines represent subjective, experiential elements; dashed lines represent brain activity levels as measured by regional cerebral blood flow using PET, or BOLD (blood-oxygen-level-dependent) signal using fMRI. DMN, default mode network; PFC, prefrontal cortex.

Finally, we observed a number of significant clusters of deactivation (REM < waking rest), nearly all of which were in lateral PFC regions, including left RLPFC and bilateral orbitofrontal cortex. These regions have been strongly implicated in top-down regulation of emotion, cognitive control, and metacognitive monitoring (e.g., Christoff and Gabrieli, 2000), and are among the key areas that become more active with a variety of effortful, top-down tasks, as compared to the resting state. This suggests a trend of decreasing PFC activity from waking thought, through MW, to dreaming (see below, and Figure 3).

### Dreaming as intensified mind wandering: evidence from first-person reports

In many ways, first-person experiences in both states are similar: dreams and spontaneous thoughts are both likely (~20–30% of reports) to contain bizarre or implausible elements, to contain positive or negative emotion (~60–80% of reports), to draw on proximal and distal memory sources, to relate strongly to subjects' current concerns, and to involve simulated social interaction. Differences are apparent in other respects, however, and we argue that each difference suggests a greater preponderance or “intensity” of a given element in dreaming. First, the sensory aspects of dreams are far more immersive and intense than during waking spontaneous thought. Waking spontaneous thoughts tend to be tinged with audiovisual aspects, which typically coincide with some level of awareness of the external environment and sensory inputs. In dreaming, conversely, external sensory inputs are almost entirely blocked, and the audiovisual content can take on the aspect of an immersive, three-dimensional simulated reality. Second, the potential for bizarre or impossible content seems not only more common but more intense in dreams, though the debate over how to measure “bizarreness” makes strong claims impossible in this respect. Third, dreams appear to be temporally extended, fairly cohesive narratives spanning several minutes or longer, whereas waking MW thoughts typically only last for several seconds (Klinger, 1978). Fourth, a recent study examining the memory sources of dreams found that a substantial amount of dream content that was traceable to waking experience (~39% of memory sources) was in fact “replay” or recall of waking *thoughts*, as opposed to perceptions or other experiences (Fosse et al., 2003), further suggesting that dreaming amplifies and intensifies waking thoughts. Taken together, these findings (as well as our meta-analytic neuroimaging results—see next section) support the idea that dreaming can be seen as an intensified version of waking spontaneous thought—or conversely, that MW during wakefulness could be seen as an attenuated, waking form of dreaming (or, as its colloquial moniker suggests, “daydreaming”) (see Figure 3).

### Neural evidence for dreaming as intensified mind wandering

To ensure a consistent picture of REM sleep brain activity, we only included in our meta-analysis studies that used relaxed wakefulness (instead of, e.g., other sleep stages) as a baseline condition. Thus the activations observed in REM sleep (Table 2) are in contrast to quiet, waking rest, which—though not directly examined in the studies in question—would very likely have resulted in spontaneous thought/MW at the subjective level, and recruited DMN brain regions. Since the observed foci of activation generally represent *t*-tests contrasting REM sleep > waking rest, it seems probable that our meta-analytic results actually represent regions showing *greater* activity during REM sleep than during DMN activation/MW. Because so many significant clusters for REM sleep activation overlapped with DMN regions, these results suggest that brain activity in REM sleep does not simply *parallel* DMN activity, but rather represents an intensified version of it (Figure 3). The finding of greater cerebral blood flow in DMN regions during REM sleep vs. probable waking DMN activity is consistent with the many qualitative, first-person results discussed above (Section First-person Reports of Content from Mind Wandering and Dreaming), which suggest that mentation during REM sleep is in many ways a longer, immersive, more intensive version of waking spontaneous thoughts and daydreams (Figure 3).

Also of interest are prefrontal cortical (PFC) regions, involved in executive processes like cognitive control and goal-directed thought. It is well known that numerous such regions, particularly the anterior cingulate cortex (ACC) and dorsolateral PFC (DLPFC), are consistently engaged by effortful, goal-directed tasks (Duncan and Owen, 2000). Though executive PFC regions are not part of the canonical DMN (Table 3; Buckner et al., 2008), more direct, online assessments of MW, using first-person reports combined with fMRI, show that executive PFC areas, alongside core DMN areas, may also be activated during MW (Christoff et al., 2009). Though MW-related activity was not observed in some other PFC regions, robust activation was found in dorsal ACC and DLPFC (Christoff et al., 2009), suggesting that executive processes may to some degree be ongoing during MW. REM sleep, in contrast, shows no such activations; indeed, we found numerous executive PFC regions to be deactivated (Table 2, Figure 1). We propose the tentative notion that waking thought, waking MW, and dream mentation may lie along a continuum of intensity with respect to executive function, as well: executive regions are most active during waking goal-directed thought, undergo a large (but probably not total) diminution during waking rest/MW, and become relatively quiescent, perhaps even actively suppressed, during REM sleep (Figure 3; see also Christoff et al., 2011).

### Putative functions of spontaneous thought during wakefulness and sleep

Numerous reviews have recently examined potential functions of spontaneous thought/DMN (Buckner et al., 2008; Klinger, 2008; Christoff et al., 2011; Andrews-Hanna, 2012) and dreaming/REM sleep (e.g., Domhoff, 2003; Deseilles et al., 2011, Ch. 6), so we offer only a brief overview of key ideas here. Despite sparse empirical data overall, discussion of functionality seems to us necessary because brains, particularly those as large as the ones possessed by *homo sapiens*, are very metabolically expensive organs to maintain, consuming an egregiously disproportionate share of the body's energy when compared to their relative mass (roughly 15–20% of the body's basal metabolic energy expenditure for a mere ~2% of its body mass: Aiello and Wheeler, 1995). The large amount of time spent in REM sleep (~1.5–2 h per night) and the prevalence of MW during wakefulness (~30–50% of waking thought) collectively suggest that a non-trivial proportion of this metabolic energy is dedicated to spontaneous thought in one form or another, inviting the question of what biological-evolutionary function the latter might serve.

One major theory is that spontaneous thought involves goal-oriented (if still somewhat “undirected”) processing of current concerns and planning for the future (Buckner et al., 2008; Klinger, 2008; Baird et al., 2011; Stawarczyk et al., 2011; Andrews-Hanna, 2012; Mooneyham and Schooler, 2013). This notion is consonant with the large amount of subjective content focused on imagined future scenarios and with the high prevalence of subjects' current concerns in content reports of both dreams and waking MW (Section First-person Reports of Content from Mind Wandering and Dreaming). On this view, a major function of the brain when not strongly occupied by external stimuli is to address current issues and plan for future events, both expected and hypothetical. This process would likely involve the recombination of episodic and semantic memories to yield plausible future scenarios, explaining in part the large proportion of past-oriented thought evident in both waking and sleeping spontaneous thought.

A second possibility, complementary to the first, is that of offline memory consolidation and reconsolidation (Christoff et al., 2011). Memory traces are known to be reactivated during REM sleep, both in terms of replay of neural activity sequences as observed with single-cell recordings in rats (Wilson and McNaughton, 1994), and reactivation of regions shown to be active during learning, as revealed by fMRI in human subjects (Maquet et al., 2000). Intriguingly, very similar results have been found during periods of wakefulness after training, again at the single-cell level in rats (Sutherland and McNaughton, 2000; Foster and Wilson, 2006) and at the regional level with fMRI in humans (Peigneux et al., 2006). Collectively, these results suggest that the subjective experiences of wakeful MW and dream mentation may represent, at least in part, the phenomenal side of an underlying brain process involving memory consolidation and reconsolidation (see Christoff et al., 2011, for a more detailed discussion).

A third idea, oft-reported anecdotally but difficult to demonstrate experimentally, is that dreams and daydreams serve to facilitate creativity, insight, and problem-solving, sometimes explained via the mechanism of “incubation” (Schredl and Erlacher, 2007; Baird et al., 2012). Though intriguing, support for this idea remains mostly anecdotal (see Introduction; also Csikszentmihalyi, 1996). Experimental studies have begun to address this question, however (e.g., Baird et al., 2012), and a recent fMRI study from our group found higher activity in DMN regions (hippocampus, parahippocampus, and inferior parietal lobule—all bilaterally) during the generation of creative artwork (Ellamil et al., 2012).

### Limitations

Several limitations of the present review and meta-analysis should be acknowledged. First and most important is the use of DMN and REM sleep brain activity as neuromarkers for MW and dreaming, respectively. Though both pairs of states are tightly coupled, as outlined in detail above, it should be stressed that they are by no means identical. We therefore anticipate that in the future, more specific neuroimaging work will directly target MW and dreaming, as distinct from DMN and REM sleep activity, respectively, both extending and improving upon the present preliminary results. This issue is considered in greater detail in the Methods section above.

Second, though the meta-analytic neural substrate of dreaming overlaps considerably with that of the DMN, many activation clusters extend beyond DMN hubs. These discrepancies may be due in part to noise attributable to the small sample size of REM studies (6 reports), but they likely also reflect real brain substrate differences between these two states. In our view, some of these differences are consonant with the aforementioned “intensity” hypothesis; others, however (e.g., minimal PCC, and total lack of IPL and LTC activations in REM sleep) may point toward either genuine differences in neural substrate, or possibly very similar levels of activity indistinguishable by subtraction contrasts (REM > waking rest, or waking rest > REM).

Third, despite many experiential and neural similarities, dreaming is predictably engaged in for long periods each night (several minutes to over an hour) throughout the sleep cycle, particularly during REM sleep (Aserinsky and Kleitman, 1953; Dement and Kleitman, 1957), whereas daydreaming tends to be more sporadic and short-lived, and is most commonly occasioned by low external task demands (Antrobus et al., 1966), among other factors (Smallwood and Schooler, 2006). The mechanisms of initiation, and/or impetus for the content reaching conscious awareness, may therefore be distinct.

Fourth, the neurochemical dynamics of REM sleep differ markedly from those of normal waking (Solms, 2002). It may be that the neurochemistry of MW and/or quiet waking rest differs from that of normal waking, and might even resemble that of REM sleep—or lie somewhere between the two. To our knowledge, this remains a largely unexplored question (though see Christoff et al., 2011, for a discussion), but it seems probable that the neurochemical basis of waking rest and/or MW will differ in important ways from the exceptional neurochemistry of REM sleep.

Fifth, all the studies in our meta-analysis of REM sleep employed PET imaging (the incredible noise created by fMRI scanners make sleep studies difficult), whereas all the studies included in the meta-analysis of Buckner et al. (2008) to identify core regions of the DMN (Table 3) employed fMRI. Although this presents the possibility of systematic confounding differences, data from both modalities is routinely pooled together in meta-analyses and reviews. Further, for the DMN at least, studies have been conducted using both modalities with similar results. Indeed, the early work (e.g., Andreasen et al., 1995; Raichle et al., 2001) upon which all subsequent investigation of the DMN has been based used exclusively PET imaging; much subsequent work with fMRI, however, has largely confirmed these early PET results (see Buckner et al., 2008), reinforcing the idea of a certain degree of comparability across these two modalities.

### Future directions

The relation between brain activity across sleep stages, the associated subjective content related to each stage, and the potential involvement of the DMN remain open and intriguing questions for future research. Interestingly, other sleep stages (beyond REM) show evidence for dream-like mentation to varying degrees, with late-night NREM2 and a brief epoch at sleep onset (NREM1) of particular interest, whereas other stages (early-night NREM2 and NREM3/4 or SWS) are associated with little subjective experience. Intriguingly, some studies have shown decreased DMN functional connectivity across various NREM sleep stages (Horovitz et al., 2009; Sämann et al., 2011), consistent with our central hypothesis; others, however, find more complex relationships among subsets of the DMN (Koike et al., 2011). Research also continues apace into functional connectivity among various other brain regions and networks (other than the DMN) throughout the sleep cycle (e.g., Horovitz et al., 2008; Larson-Prior et al., 2009), further complicating the picture.

One particular case allows for a fairly straightforward prediction, however: late-night/early-morning NREM2 laboratory awakenings give rise to more, and more dream-like, mentation reports than awakenings from NREM2 cycles early in the night (Antrobus et al., 1995; Cicogna et al., 1998). Though REM is the predominant sleep stage later in the night, presenting a potential confound, these qualitative results nonetheless suggest that late- vs. early-night NREM2 sleep may show distinctive patterns of brain activity, though to our knowledge this has yet to be examined with functional neuroimaging. Based on first-person reports and the present meta-analytic results, we hypothesize that late-night NREM2, if isolable, may show brain activity similar to the DMN and REM sleep (see also Domhoff, 2011).

Comprehensive testing of the various theoretical accounts of putative functionality for spontaneous thought and dreaming is also important. Though at least some spontaneous thoughts seem of undeniable value to individuals, there appear too to be many less-than-useful thoughts, and incoherent dreams. Future work can address this issue by exploring differential neural correlates and subjective qualities of dreams and spontaneous thoughts related to any number of factors of interest, such as creativity and planning for the future (see, e.g., Andrews-Hanna et al., 2010; Stawarczyk et al., 2011).

Clearly, much work remains to be done in elucidating the connections between MW, dreaming, REM sleep and the DMN. In particular, future work should further examine spontaneous thoughts from both wakefulness and sleep stages in the *same* subjects to allow for direct comparisons of first-person reports. Ideally, such studies could also compare DMN activity and REM sleep activation in the same subjects, as well.

Though subjective content reports have long suggested similar neural processes underlying dreaming and waking MW, here we have presented the first strong neuroimaging evidence that this is indeed the case. We hope that subsequent behavioral and neuroimaging research, ideally conducted in conjunction with detailed first-person reports, will increase our knowledge of these still poorly understood mental states, and amplify the present finding of their shared neural basis.

### Conflict of interest statement

The authors declare that the research was conducted in the absence of any commercial or financial relationships that could be construed as a potential conflict of interest.

## Appendix

**Table A1:** **Summary of included and excluded neuroimaging studies of REM sleep**.

**Study**	**Modality**	**Subjects**	**Included?**	**Reason for exclusion**
Hong et al., 1995	PET	9	N	Only epochs containing REMs/saccades analyzed
Maquet et al., 1996	PET	11	Y	–
Nofzinger et al., 1997	PET	6	Y	–
Braun et al., 1997	PET	37	Y	–
Braun et al., 1998	PET	10	Y	–
Lövblad et al., 1999	fMRI	5	N	No peak foci reported
Maquet et al., 2000	PET	5	Y	–
Peigneux et al., 2001	PET	12	Y	–
Wehrle et al., 2005	fMRI	11	N	Within-REM sleep comparisons only
Wehrle et al., 2007	fMRI	11	N	Auditory stimulation during sleep
Hong et al., 2009	fMRI	11	N	Only epochs containing REMs/saccades analyzed
Miyauchi et al., 2009	fMRI	17	N	Within-REM sleep comparisons only
Dresler et al., 2012	fMRI	4	N	Within-REM sleep comparisons only
Germain et al., 2013	PET	18	N	Clinical population (combat veterans with PTSD)

fMRI, functional magnetic resonance imaging; N, no; PET, positron emission tomography; PTSD, post-traumatic stress disorder; REM, rapid eye movement; Y, yes.
